# Supplementary material for: Pt-Based Nanostructures for Electrochemical Oxidation of CO: Unveiling the Effect of Shapes and Electrolytes
Source: Int J Mol Sci. 2022 Nov 30;23(23):15034. doi: 10.3390/ijms232315034 (PMC9737813; doi:10.3390/ijms232315034)
Supplement: Supplementary file 1 [file ijms-23-15034-s001.zip › ijms-1964986-supplementary.pdf]

Supporting information

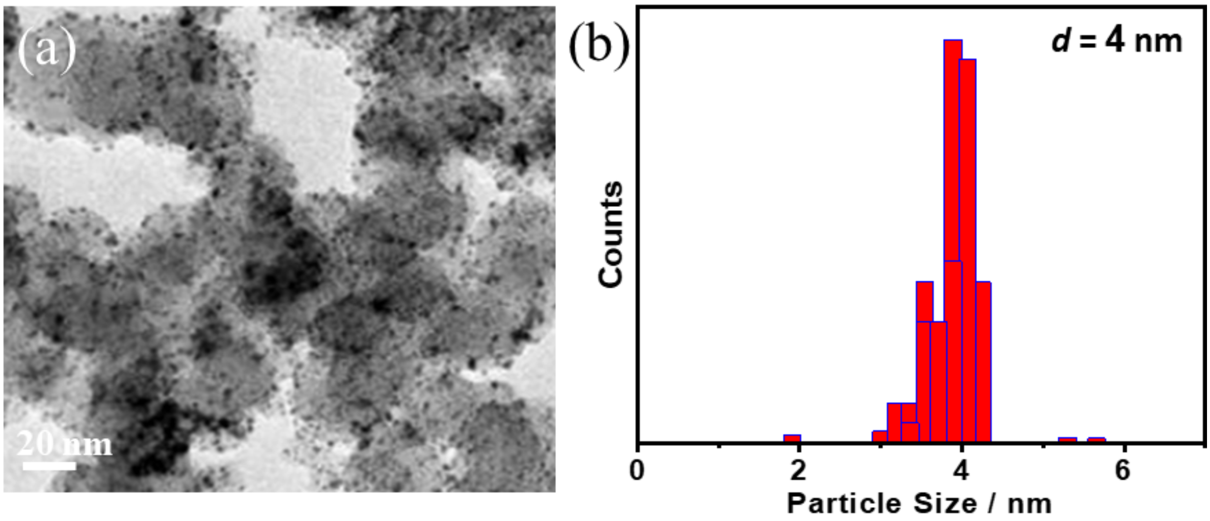

Figure S1. (a) TEM image of commercial and (b) histogram of particle size distribution of commercial Pt/C catalyst

Table S1.binding energies of Pt 4f in Pt PNDs, Pt NCs, and Pt/C catalysts.

**catalyst**	**Pt 4f $_{7/2}$**		**Pt 4f$_{5/2}$**	
	**Pt^0**	**Pt^{2+}**	**Pt^0**	**Pt^{2+}**
**Pt NDs**	71.31	72.2	74.66	75.55
**Pt NCs**	71.24	72.1	74.55	75.45
**Pt/C**	71.15	71.9	74.46	75.3

Table S2. The ECSA extracted form hydrogen under-potential adsorption/desorption (H-UPD) ($ECSA_{H-UPD}$) and CO under under-potential adsorption ($ECSA_{CO-UPD}$)

	$HClO_4$		KOH		Na_2HCO_3	
Catalsysts	$ECSA_{H-UPD}$ (m^2/g)	$ECSA_{CO-UPD}$ (m^2/g)	$ECSA_{H-UPD}$ (m^2/g)	$ECSA_{CO-UPD}$ (m^2/g)	$ECSA_{H-UPD}$ (m^2/g)	$ECSA_{CO-UPD}$ (m^2/g)
Pt NDs	59.1	34.23	66	30.71		19.52381
Pt NCs	52.2	15.23	65.2	22.18		18.85714
Pt/C	48.3/	14.38	52.5	18.28		11.2381

Table S3. Comparison of the electrochemical CO oxidation activities of our synthesized catalysts relative to previously reported catalysts.

Catalyst	Medium / Scan rate / reference electrode	Maximum Current (mA/cm^{-2}) / Voltage (V)	Ref
60 wt % Pt/C	0.5 H2SO4<br>10 mV s^{-1}<br>SHE	0.2 mA cm^{-2}<br>0.64 V	[1]
Well ordered Pt(111)	0.1 M NaOH<br>50 mV/s<br>RHE	0.5 mA cm^{-2}<br>0.8 V	[2]
PtNi multicubes	1 M KOH<br>50 mV/s<br>RHE	0.58 mA cm^{-2}<br>0.65 V	[3]
PtPd Nanodendrites	1 M KOH<br>50 mV/s<br>Ag/AgCl	5.1 mA cm^{-2}<br>-0.15 V	[4]
Pt dendrimer-encapsulated nanoparticles	0.1m $HClO_4$<br>50 mV/s<br>Hg/Hg_2SO_4	0.2 mA cm^{-2}<br>0.3 V	[5]
Pt(110)–Ru	0.5 M H_2SO_4<br>100 mV/s<br>RHE	0.025 mA<br>0.5 V	[6]
Pt/SnO_x	1 M $HClO_4$<br>20 mV s^{-1}<br>RHE	0.87 mA cm^{-2}<br>0.7 V	[7]
Pt-NbOx	0.5 M H_2SO_4<br>20 mV/s<br>RHE	0.5 mA cm^{-2}<br>0.75 V	[8]
Pt(FAM)	0.1 M H_2SO_4<br>50 mV/s<br>RHE	0.32 mA cm^{-2}<br>0.72 V	[9]
PtRu@h-BN/C	0.1 M H_2SO_4<br>20 mV/s<br>RHE	1.25 mA cm^{-2}<br>0.6 V	[10]
Pt NDs	0.1 M $HClO_4$<br>50 mV/s<br>Ag/AgCl	2.233 mA cm^{-2}<br>0.496 V	Our work
	0.5 M Na_2HCO_3<br>50 mV/s<br>Ag/AgCl	0.44mA cm^{-2}<br>0.18 V	
	0.1 M NaOH<br>50 mV/s<br>Ag/AgCl	1.71mA cm^{-2}<br>-0.228 V	

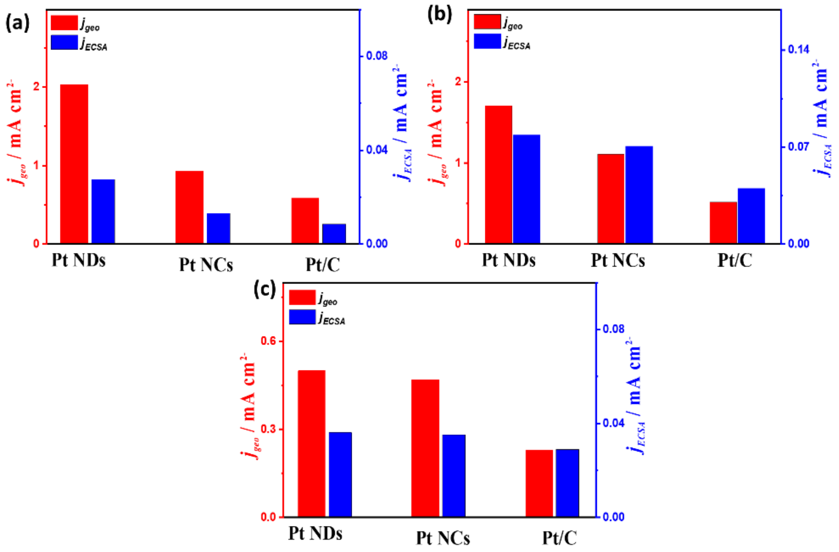

Figure S2. The current normalized by the electrode area (j_{geo}) and ECSA (j_{ECSA}) of different catalysts in (a) $HClO_4$, (b) KOH, and (c) $NaHCO_3$ electrolytes

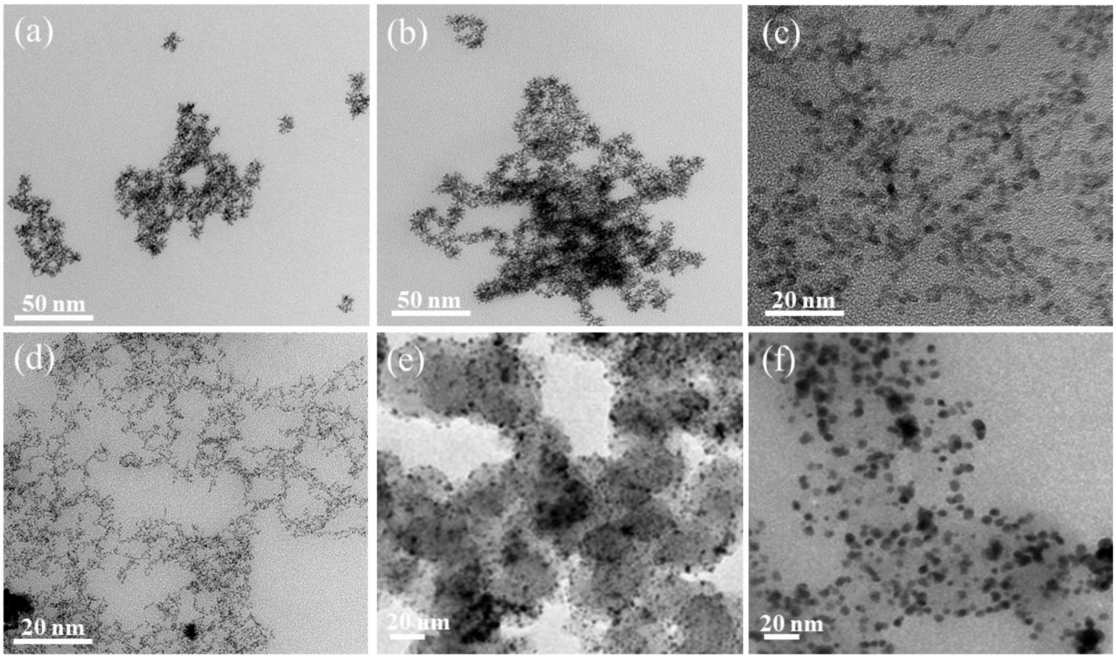

Figure S3. TEM image before and after stability tests of (a-b) Pt PNDs, (c-d) Pt NCs, and (e-f) Pt/C, respectively

References

1. McPherson, I.J.; Ash, P.A.; Jones, L.; Varambhia, A.; Jacobs, R.M.; Vincent, K.A. Electrochemical CO oxidation at platinum on carbon studied through analysis of anomalous in situ IR spectra. *The Journal of Physical Chemistry C* **2017**, *121*, 17176-17187.
2. Spendelow, J.; Goodpaster, J.; Kenis, P.; Wieckowski, A. Mechanism of CO oxidation on Pt (111) in alkaline media. *The Journal of Physical Chemistry B* **2006**, *110*, 9545-9555.
3. Wu, F.; Eid, K.; Abdullah, A.M.; Niu, W.; Wang, C.; Lan, Y.; Elzatahry, A.A.; Xu, G. Unveiling one-pot template-free fabrication of exquisite multidimensional PtNi multicube nanoarchitectonics for the efficient electrochemical oxidation of ethanol and methanol with a great tolerance for CO. *ACS applied materials & interfaces* **2020**, *12*, 31309-31318.
4. Eid, K.; Ahmad, Y.H.; Yu, H.; Li, Y.; Li, X.; AlQaradawi, S.Y.; Wang, H.; Wang, L. Rational one-step synthesis of porous PtPdRu nanodendrites for ethanol oxidation reaction with a superior tolerance for CO-poisoning. *Nanoscale* **2017**, *9*, 18881-18889.
5. Weir, M.G.; Myers, V.S.; Frenkel, A.I.; Crooks, R.M. In situ X-ray absorption analysis of~ 1.8 nm dendrimer-encapsulated Pt nanoparticles during electrochemical CO oxidation. *ChemPhysChem* **2010**, *11*, 2942-2950.
6. Davies, J.C.; Hayden, B.E.; Pegg, D.J. The electrooxidation of carbon monoxide on ruthenium modified Pt (110). *Electrochimica acta* **1998**, *44*, 1181-1190.
7. Matsui, T.; Fujiwara, K.; Okanishi, T.; Kikuchi, R.; Takeguchi, T.; Eguchi, K. Electrochemical oxidation of CO over tin oxide supported platinum catalysts. *Journal of power sources* **2006**, *155*, 152-156.
8. Ueda, A.; Yamada, Y.; Ioroi, T.; Fujiwara, N.; Yasuda, K.; Miyazaki, Y.; Kobayashi, T. Electrochemical oxidation of CO in sulfuric acid solution over Pt and PtRu catalysts modified with TaOx and NbOx. *Catalysis today* **2003**, *84*, 223-229.
9. Ciapina, E.G.; Santos, S.F.; Gonzalez, E.R. The electrooxidation of carbon monoxide on unsupported Pt agglomerates. *Journal of Electroanalytical Chemistry* **2010**, *644*, 132-143.
10. Sun, M.; Lv, Y.; Song, Y.; Wu, H.; Wang, G.; Zhang, H.; Chen, M.; Fu, Q.; Bao, X. CO-tolerant PtRu@ h-BN/C core–shell electrocatalysts for proton exchange membrane fuel cells. *Applied Surface Science* **2018**, *450*, 244-250.
